# Potential drug-drug interactions among pneumonia patients: do these matter in clinical perspectives?

**DOI:** 10.1186/s40360-019-0325-7

**Published:** 2019-07-26

**Authors:** Sidra Noor, Mohammad Ismail, Zahid Ali

**Affiliations:** Department of Pharmacy, University of Peshawar, Peshawar, Khyber Pakhtunkhwa Pakistan

**Keywords:** Pneumonia, Patient safety, Pneumonia therapy, Potential drug-drug interactions, Clinical relevance, Polypharmacy

## Abstract

**Background:**

Pneumonia patients are usually hospitalized due to severe nature of the disease or for the management of comorbid illnesses or associated symptoms. Such patients are prescribed with multiple medications which increase the likelihood of potential drug-drug interactions (pDDIs). Therefore, in this study the prevalence, levels (severity and documentation), predictors (risk factors), and clinical relevance of pDDIs among inpatients diagnosed with pneumonia have been investigated.

**Methods:**

Clinical records of 431 hospitalized patients with pneumonia were checked for pDDIs using drug interactions screening software (Micromedex-DrugReax). Odds-ratios for predictors were calculated using logistic regression analysis. Clinical relevance of pDDIs was assessed by evaluation of patients’ clinical profiles for potential adverse outcomes of the most frequent pDDIs. Abnormal patients’ signs/symptoms and laboratory investigations indicating adverse outcomes of interactions were reported.

**Results:**

Of total 431 profiles, pDDIs were reported in 73.1%. Almost half of the profiles were having major-pDDIs (53.8%). Total number of pDDIs were 1318, of which 606 were moderate- and 572 were major-pDDIs. Patient’s profiles identified with the most frequent interactions were presented with signs, symptoms, and abnormalities in labs indicating decrease therapeutic response, electrolyte abnormalities, hypoglycemia, bleeding, hepatotoxicity, and hypertension. These adverse events were more prevalent in patients taking higher doses of the interacting drugs as compared to lower doses. Logistic regression analysis revealed significant association for major-pDDIs with 6–10 prescribed medicines (OR = 26.1; *p* = 0.002), > 10 prescribed medicines (OR = 144; *p* <  0.001), and tuberculosis (OR = 8.2; *p* = 0.004).

**Conclusions:**

PDDIs are highly prevalent in patients with pneumonia. Most frequent and clinically important pDDIs need particular attention. Polypharmacy and tuberculosis increase the risk of pDDIs. Identifying patients more at risk to pDDIs and careful monitoring of pertinent signs/symptoms and laboratory investigations are important measures to reduce pDDIs and their related adverse consequences.

## Background

Worldwide, pneumonia remains the leading cause for childhood mortality and adult hospitalization, regardless of progresses in the management and preventive policies [1]. According to World Health Organization, in 2015 pneumonia causes death for approximately 920,136 children, accounting for 16% of all deaths of children younger than 5 years [2]. Pneumonia is considered as one of the contributing factors causing burden on health care system [3].

Pneumonia patients are usually hospitalized due to severe nature of the disease or for the management of comorbid illnesses or associated symptoms. The leading comorbidities of patients with pneumonia include diabetes mellitus, cerebrovascular disease, chronic lung disease, chronic kidney disease, and dementia [4]. During hospitalization such patients are prescribed with antipyretics, antitussives, antibiotics, and antihistamines [5]. Apart from the use of aforementioned drugs, such patients are prescribed with a large number of other drugs for the management of associated symptoms and comorbid illnesses [6]. There is an increased risk of drug-drug interactions (DDIs) with simultaneous use of multiple drugs. DDIs may lead to alteration in the pharmacokinetic parameters or pharmacodynamic profile of drugs [7, 8]. Many of the negative clinical consequences such as decreased or abolished clinical effectiveness, adverse drug reactions (ADRs), toxicity, hospitalization, and prolonged hospital stay are attributed by DDIs [9]. DDIs lead to 20–30% of adverse effects, of which 1–2% are life-threatening and 70% need clinical intervention [10]. Studies have usually addressed the issue of potential DDIs (pDDIs) either in a general way or on the basis of clinical specialties such as geriatrics [8], internal medicine [11], oncology [12], psychiatry [13], and cardiology [14]. Despite being one of the most frequent causes of hospitalization [15], DDIs specifically among hospitalized patients with pneumonia in clinical settings remain unaddressed. Therefore, particular attention is needed in order to conduct studies regarding pDDIs and their clinical relevance among hospitalized patients with pneumonia. Subsequently, such studies will help health care professionals to manage pDDIs and reduce their associated consequences, improve patients’ safety, and bring positive clinical outcomes.

Therefore, in this study the prevalence, levels (severity and documentation), predictors (risk factors), and clinical relevance of pDDIs among inpatients diagnosed with pneumonia have been investigated.

## Methods

### Study design and settings

The present study was carried out in internal medicine wards at tertiary care settings (KTH: Khyber Teaching Hospital and HMC: Hayatabad Medical Complex) of the provincial capital (KPK, Khyber Pakhtunkhwa) using a cross-sectional retrospective design. Khyber Teaching Hospital is located at the main university road of the provincial capital, while Hayatabad Medical Complex is located in the Town III of the city. Khyber Teaching Hospital delivers health care and referral services to the residents of Peshawar University Town and adjacent areas, while Hayatabad Medical Complex provides services to the western parts of Peshawar, its neighboring areas, and patients coming from Afghanistan. Both the hospitals are lacking clinical pharmacy services at the level of the wards. Moreover, software-based drug interactions screening programs are lacking in both the hospitals. Patients’ data are maintained in the predesigned charts and kept in the main record room of the hospitals.

### Inclusion and exclusion criteria

The study included inpatients diagnosed with pneumonia during the study period (from 1-Jan-15 to 31-Dec-16), and of either gender and age. Patients’ profiles lacking relevant data required for the study were excluded.

### Sample size calculation

The calculated sample size was 383 based on the anticipated prevalence of 52.8% [15], 95% confidence level, and 5% margin of error [16]. However, total 431 patients were eligible for inclusion during the study period (from January 2015 to December 2016); therefore, all were included.

### Data source

Administrative permission was obtained from both the hospitals for the access of patients’ clinical record. Convenient sampling technique was used for collecting the following data: patients’ demographics, hospital admissions and discharge dates, diagnoses, comorbidities, medications therapy at the hospital, signs/symptoms, and laboratory tests.

### Screening for pDDIs

Micromedex Drug-Reax® [17] was used for checking patients’ medications profiles for the identification of DDIs. This software classifies DDIs according to severity- and documentation-levels [17]. The detail description of these levels is available elsewhere [18–20].

Prevalence of pDDIs as well as prevalence of severity-levels were identified. List of the most frequent (widespread) and clinically important pDDIs was provided. The list also includes potential adverse consequences and levels (severity as well as documentation) of such pDDIs.

### Clinical relevance of pDDIs

Clinical relevance of pDDIs was assessed by evaluating each patient’s profile for potential adverse outcomes of top-10 pDDIs. Abnormal patients’ signs/symptoms and laboratory tests indicating adverse outcomes of interactions were reported. The clinical features were stratified based on dose differences of interacting drugs. The following cut off points were used for defining higher daily doses, furosemide: ≥60 mg; hydrocortisone: ≥500 mg; aspirin: ≥150 mg; insulin: > 20 units; isoniazid: ≥150 mg; rifampin: ≥300 mg; calcium containing products: ≥1 g; ceftriaxone: ≥4 g; pyrazinamide: ≥500 mg; ramipril: ≥5 mg; albuterol: ≥15 mg/3 ml; bisoprolol: ≥5 mg. In this study, adverse outcomes were defined as follows, increased blood urea nitrogen (BUN): BUN ≤20 mg/dL; increased serum creatinine: serum creatinine > 1.06 mg/dL; hypernatremia: serum sodium > 145 mmol/L; hyponatremia: serum sodium < 135 mmol/L; hyperkalemia: serum potassium > 5.5 mmol/L; hypokalemia: serum potassium < 3.5 mmol/L; hyperchloremia: serum chloride > 105 mmol/L; hypertension: systolic blood pressure (BP) > 130 mmHg and/or diastolic BP > 90 mmHg; hypotension: systolic BP < 80 mmHg and/or diastolic BP < 50 mmHg; bradycardia: heart rate < 70 beats/min; tachycardia: heart rate > 100 beats/min; increased activated partial thromboplastin time (APTT): APTT > 35.5 s; increased prothrombin time (PT): PT > 15.5 s; increased international normalized ratio (INR): INR > 1.2; decreased platelets: platelets count < 150,000/μL; hypoglycemia: random blood sugar < 80 mg/dL or fasting blood sugar < 70 mg/dL; increased alkaline phosphatase: > 126 U/L; increased serum bilirubin: > 1 mg/dL; increased alanine aminotransferase: > 59 U/L (male), > 36 U/L (female); leukocytosis: total leukocyte count > 11,000/μL.

### Statistical analysis

Data were presented in frequencies and percentages form and where appropriate median (interquartile range (IQR)) was also provided. Binary logistic regression analysis (both univariate as well as multivariate) with enter method was applied to identify association of various predictors with all interactions as well as major interactions. Presence of drug interactions (overall or major) was taken as dependent variable. Patients characteristics such as gender, age, prescribed medicines, hospitalization, and comorbidities were independent variables in the model. Odds ratios (OR) and 95% confidence intervals (CI) were calculated to identify the strength of association of each independent variable with pDDIs. Multivariate analyses were carried out for variables with a univariate *p*-value of ≤0.15. *P*-value of ≤0.05 was considered statistically significant. All the data were statistically analyzed using SPSS-v23.

## Results

### Patients’ general characteristics

Patients’ demographics and comorbidities are shown in Table 1. Of total study subjects, 51% were males. The median number of prescribed drugs was 11 (8–14) and median hospital stay was 4 days (3–6). Majority of the studied patients were aged ≥41 years (85.3%). Most were prescribed with > 10 drugs (52%). Most frequent hospitalization was ≥3 days (81.7%). Hypertension (*n* = 220), diabetes mellitus (120), stroke (120), and chronic obstructive pulmonary disease (37) were the four leading comorbidities of the studied patients.

**Table 1:** General characteristics of study patients (*n* = 431)

Characteristic	Patients: n (%^a^)
Gender	
Male	220 (51)
Female	211 (49)
Age (years)	
≤40	63 (14.6)
41–60	176 (40.8)
>60	192 (44.5)
Median (IQR)	60 (50–70)
Drugs prescribed per patient	
≤5	42 (9.7)
6–10	165 (38.3)
>10	224 (52)
Median (IQR)	11 (8–14)
Hospital stay (days)	
≤2	79 (18.3)
3–4	143 (33.2)
>4	209 (48.5)
Median (IQR)	4 (3–6)
Number of comorbidities	
No comorbidities	24 (5.6)
1–2	170 (39.4)
3–4	127 (29.4)
5	10 (2.3)
Comorbidities	
Hypertension	220 (51)
Diabetes mellitus	120 (27.8)
Stroke	120 (27.8)
Chronic obstructive pulmonary disease	37 (8.6)
Ischemic heart disease	36 (8.3)
Urinary tract infection	34 (7.9)
Tuberculosis	25 (5.8)
Hepatitis	24 (5.6)
Chronic kidney disease	20 (4.6)
Asthma	16 (3.7)
Congestive cardiac failure	14 (3.2)
Post tuberculosis bronchiectasis	12 (2.8)
Malaria	10 (2.3)
Decompensated chronic liver disease	10 (2.3)
Left ventricular failure	9 (2.1)
Miscellaneous	167 (38.7)

*IQR* Interquartile range

^a^Percentage was calculated out of total number of patients i.e., 431

### Prevalence and levels of pDDIs

Table 2 presents prevalence and levels of pDDIs. Of total 431 pneumonia patients, pDDIs were identified in 315 (73.1%) patients. In 22.7% patients, > 4 pDDIs per patient were found. Based on severity-wise prevalence, 53.8% patients were presented with major-pDDIs, while 51.5% with moderate-pDDIs. Patients with contraindicated- and minor-pDDIs were observed in a low frequency. The total recorded pDDIs were categorized based on the levels of severity and documentation. Total number of pDDIs were 1318, of which 606 were moderate- and 572 were major-pDDIs. According to documentation-levels, 690 were fair- and 491 were good-type.

**Table 2:** Prevalence and levels of potential drug-drug interactions

PDDIs	Patients: n (%)
Prevalence of pDDIs^a^	
Overall prevalence of pDDIs	315 (73.1)
Number of pDDIs per patient	
1–2	141 (32.7)
3–4	76 (17.6)
>4	98 (22.7)
Severity-wise prevalence of pDDIs	
Contraindicated	47 (10.9)
Major	232 (53.8)
Moderate	222 (51.5)
Minor	74 (17.1)
Levels of pDDIs^b^	
Severity-levels	
Contraindicated	50 (3.8)
Major	572 (43.4)
Moderate	606 (46)
Minor	90 (6.8)
Documentation-levels	
Excellent	137 (10.4)
Good	491 (37.2)
Fair	690 (52.3)

*PDDIs* Potential drug-drug interactions

^a^Percentage was calculated out of total number of patients i.e., 431

^b^Percentage was calculated out of total number of potential drug-drug interactions i.e., 1318

Overall-prevalence is the occurrence of at least one pDDI irrespective of severity type. Total number of pneumonia patients were 431. Therefore, overall-prevalence of pDDIs was 73.1% (315 out of 431)

### Risk factors of pDDIs

Results regarding exposure to all types- and major-pDDIs stratified with respect to patient’s characteristics are presented in Table 3. PDDIs were more common in males as compared to females. Moreover, pDDIs were more frequently found in patients with an age range of 31 to 60 years, prescribed with > 10 medicines, and > 4 days hospitalization. Additionally, concerning comorbidities, pDDIs were mostly reported in hypertension, diabetes mellitus, stroke, and ischemic heart disease.

**Table 3:** Exposure to all types- and major-pDDIs stratified with respect to patients’ characteristics

Patient’s characteristics	All types of interactions	Only major interactions
	Patients: n (%)	Patients: n (%)
Gender		
Male	162 (51.4)	121 (52.2)
Female	153 (48.6)	111 (47.8)
Age (years)		
≤30	19 (6)	13 (5.6)
31–60	154 (48.9)	116 (50)
>60	142 (45.1)	103 (44.4)
Drugs prescribed per patient		
≤5	7 (2.2)	1 (0.4)
6–10	103 (32.7)	60 (25.9)
>10	205 (65.1)	171 (73.7)
Hospital stay (days)		
≤2	47 (14.9)	28 (12.1)
3–4	97 (30.8)	71 (30.6)
>4	171 (54.3)	133 (57.3)
Comorbidities		
Hypertension	173 (54.9)	128 (55.2)
Diabetes mellitus	102 (32.4)	77 (33.2)
Stroke	98 (31.1)	75 (32.3)
Chronic obstructive pulmonary disease	25 (7.9)	13 (5.6)
Ischemic heart disease	30 (9.5)	25 (10.8)
Urinary tract infection	25 (7.9)	21 (9.1)
Tuberculosis	22 (7)	21 (9.1)
Hepatitis	16 (5.1)	11 (4.7)
Chronic kidney disease	16 (5.1)	9 (3.9)
Asthma	14 (4.4)	10 (4.3)

Table 4 presents ORs with corresponding 95%CIs for pDDIs of all types using univariate model. The results were significant with patient’s age 31–60 years (OR = 3.5; *p* <  0.001) & > 60 years (OR = 3; *p* = 0.002), prescribed with 6–10 medicines (OR = 8.3; *p* <  0.001), > 10 medicines (OR = 53.9; *p* <  0.001), and > 4 days hospitalization (OR = 3.1; *p* <  0.001). Moreover, concerning comorbidities, significant association of all types-pDDIs was found with hypertension (OR = 1.8; *p* = 0.008), diabetes mellitus (OR = 2.6; *p* = 0.001), stroke (OR = 1.9; *p* = 0.01), ischemic heart disease (OR = 1.9; *p* = 0.15), and tuberculosis (OR = 2.8; *p* = 0.09).

**Table 4:** Logistic regression analysis based on exposure to all types- and major-pDDIs

Variables	All types-pDDIs^a^				Major-pDDIs^b^			
	Univariate analysis		Multivariate analysis		Univariate analysis		Multivariate analysis	
	OR (95% CI)	*p*-value	OR (95% CI)	*p*-value	OR (95% CI)	*p*-value	OR (95% CI)	*p*-value
Gender								
Male	Reference		–		Reference		–	
Female	0.9 (0.6–1.4)	0.8	–	–	0.9 (0.6–1.3)	0.6	–	–
Age (Years)								
≤30	Reference		Reference		Reference		Reference	
31–60	3.5 (1.7–7.2)	< 0.001	1.9 (0.7–4.7)	0.2	2.8 (1.3–5.7)	0.006	2.1 (0.8–5.7)	0.1
>60	3 (1.5–6)	0.002	1.5 (0.6–3.9)	0.4	2.3 (1.1–4.8)	0.02	1.7 (0.6–4.5)	0.3
Drugs prescribed								
≤5	Reference		Reference		Reference		Reference	
6–10	8.3 (3.5–20)	< 0.001	7.3 (2.9–18.4)	< 0.001	23.4 (3.1–174.7)	0.002	26.1 (3.3–210)	0.002
>10	53.9 (21–138)	< 0.001	43.3 (15.6–120)	< 0.001	132.3 (18–985)	< 0.001	144 (18–1177)	< 0.001
Hospital stay (days)								
≤2	Reference		Reference		Reference		Reference	
3–4	1.4 (0.8–2.5)	0.2	0.7 (0.3–1.3)	0.3	1.8 (1–3.2)	0.04	0.8 (0.4–1.6)	0.5
>4	3.1 (1.7–5.4)	< 0.001	0.9 (0.5–2)	0.9	3.2 (1.9–5.5)	< 0.001	0.9 (0.5–1.9)	0.9
Comorbidities								
Hypertension	1.8 (1.2–2.8)	0.008	0.9 (0.5–1.7)	0.9	1.4 (0.9–2.1)	0.07	0.9 (0.5–1.5)	0.5
Diabetes mellitus	2.6 (1.5–4.5)	0.001	1.8 (0.9–3.5)	0.08	1.8 (1.2–2.8)	0.008	1.2 (0.7–2.1)	0.5
Stroke	1.9 (1.1–3.3)	0.01	1.5 (0.8–2.9)	0.19	1.6 (1.1–2.5)	0.03	1.4 (0.8–2.5)	0.2
Chronic obstructive pulmonary disease	0.7 (0.4–1.5)	0.4	–	–	0.4 (0.2–0.9)	0.02	0.4 (0.2–0.9)	0.03
Ischemic heart disease	1.9 (0.8–4.8)	0.15	1.3 (0.5–3.6)	0.6	2.1 (0.9–4.3)	0.05	1.8 (0.8–4.4)	0.2
Urinary tract infection	1 (0.5–2.3)	0.9	–	–	1.4 (0.7–2.9)	0.3	–	–
Tuberculosis	2.8 (0.8–9.6)	0.09	3.7 (0.9–16.2)	0.08	4.8 (1.6–14.4)	0.004	8.2 (1.9–34.7)	0.004
Hepatitis	0.7 (0.3–1.7)	0.5	–	–	0.7 (0.3–1.6)	0.4	–	–
Chronic kidney disease	1.5 (0.5–4.6)	0.5	–	–	0.7 (0.3–1.7)	0.4	–	–
Asthma	2.7 (0.6–11.8)	0.2	–	–	1.4 (0.5–4.1)	0.5	–	–

*pDDIs* Potential drug-drug interactions

^a^Hosmer–Lemeshow goodness-of-fit test: *p* = 0.5

^b^Hosmer–Lemeshow goodness-of-fit test: *p* = 0.7

In multivariate model, all types of pDDIs were significantly associated with 6–10 prescribed medicines (OR = 7.3; *p* <  0.001), and > 10 prescribed medicines (OR = 43.3; *p* <  0.001) (Table 4).

Table 4 further presents logistic regression analysis for exposure to major-pDDIs. The univariate logistic regression analysis showed significant association with patients age 31–60 years (OR = 2.8; *p* = 0.006) & > 60 years (OR = 2.3; *p* = 0.02), prescribed with 6–10 medicines (OR = 23.4; *p* = 0.002), > 10 medicines (OR = 132.3; *p* <  0.001), and hospital stay of 3–4 days (OR = 1.8; *p* = 0.04) & > 4 days (OR = 3.2; *p* <  0.001). Moreover, concerning comorbidities, significant association of major-pDDIs was found with hypertension (OR = 1.4; *p* = 0.07), diabetes mellitus (OR = 1.8; *p* = 0.008), stroke (OR = 1.6; *p* = 0.03), ischemic heart disease (OR = 2.1; *p* = 0.05), and tuberculosis (OR = 4.8; *p* = 0.004).

In multivariate model, association of major-pDDIs remained significant with 6–10 prescribed medicines (OR = 26.1; *p* = 0.002), > 10 prescribed medicines (OR = 144; *p* <  0.001), and tuberculosis (OR = 8.2; *p* = 0.004) (Table 4).

### Widespread interacting drug pairs

Most commonly identified and clinically important pDDIs are shown in Table 5. Potential adverse consequences of such interactions were nephrotoxicity, hypokalemia, bleeding, hypoglycemia or hyperglycemia, hepatotoxicity, reduction in therapeutic effectiveness, hypertension, hypotension, gastrointestinal ulceration, QT interval prolongation, and hyperkalemia.

**Table 5:** Description of the top-20 and clinically important potential drug–drug interactions in patients with pneumonia

Interacting pairs	Frequency	Severity	Documentation	Potential adverse outcomes
Aspirin – Furosemide	40	Major	Good	Reduced diuretic effectiveness and possible nephrotoxicity
Furosemide – Hydrocortisone	39	Moderate	Fair	Hypokalemia
Aspirin – Clopidogrel	37	Major	Fair	Increased risk of bleeding
Aspirin – Insulin	33	Moderate	Fair	Hypoglycemia
Isoniazid – Rifampin	33	Major	Good	Hepatotoxicity
Calcium containing products – Ceftriaxone	33	Contraindicated	Good	Formation of ceftriaxone-calcium precipitates and is contraindicated in neonates
Pyrazinamide – Rifampin	32	Major	Good	Hepatotoxicity
Aspirin – Ramipril	28	Moderate	Fair	Decreased ramipril effectiveness
Albuterol – Furosemide	28	Moderate	Fair	ECG changes or hypokalemia
Aspirin – Bisoprolol	23	Moderate	Good	Increased blood pressure
Furosemide – Ramipril	23	Moderate	Good	Postural hypotension (first dose)
Clarithromycin – Dexamethasone	23	Major	Fair	Decrease clarithromycin exposure and increased dexamethasone exposure
Aspirin – Dexamethasone	21	Moderate	Good	Increased risk of gastrointestinal ulceration and subtherapeutic aspirin serum concentrations
Aspirin – Nitroglycerin	20	Moderate	Good	Increase in nitroglycerin concentrations and additive platelet function depression
Clopidogrel – Esomeprazole	17	Major	Excellent	Reduced plasma concentrations of clopidogrel active metabolite and reduced antiplatelet activity
Azithromycin – Moxifloxacin	16	Major	Fair	Increased risk of QT-interval prolongation
Aspirin – Spironolactone	16	Major	Good	Reduced diuretic effectiveness, hyperkalemia, or possible nephrotoxicity
Clopidogrel – Omeprazole	13	Major	Excellent	Reduced plasma concentrations of clopidogrel active metabolite and reduced antiplatelet activity
Omeprazole – Rifampin	13	Moderate	Fair	Decreased omeprazole plasma concentrations
Ramipril – Spironolactone	13	Major	Good	Hyperkalemia

### Clinical relevance of pDDIs

Prescribed doses and administration frequencies of the interacting drugs are shown in Table 6. Drugs were given in a variety of the doses and administration frequencies. Following interacting drugs were prescribed with lower doses such as: aspirin, furosemide, clopidogrel, isoniazid, rifampin, pyrazinamide, calcium containing products, ceftriaxone, ramipril, and bisoprolol. While, following drugs were prescribed with higher doses such as: hydrocortisone, insulin, and albuterol. Lower doses of the interacting drugs were more frequent as compared to higher doses.

**Table 6:** Prescribed drugs’ doses of the top-10 interactions

Interacting pair	Dose categories^a^	Prescribed dose regimen	Number of patients
Aspirin + Furosemide	Low + Low	75 mg OD + 40 mg OD	17
	Low + High	75 mg OD + 60 mg OD	10
	Low + Low	75 mg OD + 20 mg OD	5
	Low + High	75 mg OD + 100 mg OD	3
	Low + High	75 mg OD + 40 mg BD	2
	Low + High	75 mg OD + 60 mg BD	2
	Low + High	75 mg OD + 80 mg OD	1
Furosemide + Hydrocortisone	Low + High	40 mg OD + 100 mg QID	5
	High + High	80 mg OD + 100 mg QID	4
	Low + Low	40 mg OD + 250 mg OD	3
	High + Low	60 mg OD + 50 mg QID	3
	High + High	60 mg OD + 100 mg QID	3
	Low + High	20 mg OD + 100 mg QID	3
	Low + High	20 mg OD + 500 mg OD	2
	Low + Low	40 mg OD + 50 mg QID	2
	High + Low	60 mg OD + 250 mg OD	2
	High + Low	40 mg BD + 50 mg BD	1
	Low + High	40 mg OD + 250 mg TDS	1
	High + High	60 mg OD + 500 mg TDS	1
	High + Low	60 mg BD + 100 mg TDS	1
	Low + Low	40 mg OD + 100 mg BD	1
	High + Low	60 mg OD + 100 mg BD	1
	High + High	80 mg BD + 100 mg QID	1
	High + Low	60 mg BD + 250 mg OD	1
	High + Low	80 mg OD + 250 mg OD	1
	High + High	100 mg OD + 100 mg QID	1
	High + High	80 mg OD + 500 mg QID	1
	High + Low	60 mg BD + 50 mg TDS	1
Aspirin + Clopidogrel	Low + Low	75 mg OD + 75 mg OD	35
	High + Low	150 mg OD + 75 mg OD	1
	High + Low	300 mg OD + 75 mg OD	1
Aspirin + Insulin	Low + High	75 mg OD + 20–40 units/day	21
	Low + High	75 mg OD + > 40 units/day	6
	Low + Low	75 mg OD + < 20 units/day	5
	High + Low	150 mg OD + 20 units/day	1
Isoniazid + Rifampin	Low + Low	75 mg OD + 150 mg OD	27
	High + High	150 mg OD + 300 mg OD	6
Calcium containing products + Ceftriaxone	Low + Low	200 mg/L OD + 2 g OD ATD	7
	Low + High	200 mg/L BD + 2 g BD ATD	5
	Low + Low	200 mg/L BD + 2 g OD ATD	4
	High + Low	1 g OD + 2 g OD ATD	4
	Low + Low	200 mg/L OD + 1 g BD ATD	3
	Low + Low	200 mg/L TDS + 2 g OD ATD	2
	High + High	1 g OD + 2 g BD ATD	1
	Low + Low	200 mg/L BD + 1 g BD ATD	1
	Low + Low	200 mg/L OD + 1 g OD ATD	1
	Low + Low	200 mg/L BD + 1 g OD ATD	1
	Low + High	200 mg/L BD + 3 g BD ATD	1
	Low + High	200 mg/L OD + 4 g OD ATD	1
	High + High	1250 mg BD + 2 g BD ATD	1
	High + Low	1250 mg OD + 2 g OD ATD	1
Pyrazinamide + Rifampin	Low + Low	400 mg OD + 150 mg OD	28
	High + High	500 mg OD + 300 mg OD	4
Aspirin + Ramipril	Low + Low	75 mg OD + 2.5 mg OD	20
	Low + High	75 mg OD + 5 mg OD	4
	Low + High	75 mg OD + 10 mg OD	3
	Low + Low	75 mg OD + 1.25 mg OD	1
Albuterol + Furosemide	High + Low	5 mg/ml TDS + 40 mg OD	13
	High + High	5 mg/ml TDS + 60 mg OD	8
	High + Low	5 mg/ml TDS + 20 mg OD	4
	High + High	5 mg/ml TDS + 40 mg BD	1
	Low + Low	50mcg/actuation TDS + 40 mg OD	1
	Low + High	2 mg OD + 80 mg BD	1
Aspirin + Bisoprolol	Low + Low	75 mg OD + 2.5 mg OD	14
	Low + High	75 mg OD + 5 mg OD	7
	High + High	300 mg OD + 5 mg OD	1
	High + Low	150 mg OD + 2.5 mg OD	1

*OD* Once a day, *BD* Twice a day, *QID* Four times a day, *TDS* Three times a day, *ATD* Alternate day

^a^The following cut off points were used for defining higher daily doses, furosemide: ≥60 mg; hydrocortisone: ≥500 mg; aspirin: ≥150 mg; insulin: > 20 units; isoniazid: ≥150 mg; rifampin: ≥300 mg; calcium containing products: ≥1 g; ceftriaxone: ≥4 g; pyrazinamide: ≥500 mg; ramipril: ≥5 mg; albuterol: ≥15 mg/3 ml; bisoprolol: ≥5 mg

Table 7 shows pertinent clinical features (signs/symptoms and laboratory tests) in lower and higher doses groups for top-10 pDDIs. Clinical manifestations suggesting low drug’s efficacy and electrolytes abnormalities were found in patients with the interactions; aspirin + furosemide, calcium containing products + ceftriaxone, and aspirin + ramipril. These features were highly reported among patients with higher doses of furosemide, ceftriaxone, and ramipril. In patients with the interactions furosemide + hydrocortisone and albuterol + furosemide; signs/symptoms of hypokalemia such as tachycardia, constipation, confusion, irregular heart rate, nausea, and vomiting were observed. The signs/symptoms of hypokalemia were highly prevalent among low dose groups of furosemide + hydrocortisone, and high dose groups of furosemide + albuterol. Signs/symptoms and abnormalities in labs suggesting bleeding were found in patients with the interaction, aspirin + clopidogrel. Such patients were prescribed more frequently with low doses of both clopidogrel and aspirin. Signs/symptoms and abnormalities in labs indicating hypoglycemia were more prevalent in patients with the interaction aspirin + insulin and prescribed with high doses of the insulin. Moreover, signs/symptoms and abnormalities in labs suggesting hepatotoxicity were more prevalent among patients with the interactions; isoniazid + rifampin, pyrazinamide + rifampin, and prescribed with high doses of these interacting drugs. Additionally, signs/symptoms of HTN were more frequently reported among high dose groups of aspirin + bisoprolol. Monitoring/management guidelines for top-10 pDDIs are also provided in Table 7 [17, 21].

**Table 7:** Clinical relevance, dose considerations, and monitoring/management guidelines of top-10 pDDIs in patients with pneumonia

Interactions^a^	Dose categories^a^	Signs and symptoms and Laboratory abnormalities^b^	Patients: n (%^c^)	Monitoring/management guidelines
Aspirin – Furosemide (40)	Low + Low (21)	Increased BUN	14 (66.7)	Monitoring of aspirin toxicity and renal function. Response of diuretic should be checked mainly anti-hypertensive effects. High doses are generally not recommended. Alternative may be considered where possible.
		Increased serum creatinine	10 (47.6)	
		Hyponatremia	7 (33.3)	
		Hypertension	6 (28.6)	
		Dyspnea	6 (28.6)	
		Confusion	5 (23.8)	
		Drowsiness	4 (19)	
		Edema	2 (9.5)	
		Hypokalemia	2 (9.5)	
		Hyperchloremia	1 (4.8)	
		Orthopnea	1 (4.8)	
		Chest pain	1 (4.8)	
		Nausea	1 (4.8)	
		Coma	1 (4.8)	
	Low + High (19)	Increased BUN	16 (84.2)	
		Hypertension	13 (68.4)	
		Increased serum creatinine	11 (57.9)	
		Dyspnea	7 (36.8)	
		Hyperchloremia	6 (31.6)	
		Chest pain	5 (26.3)	
		Orthopnea	5 (26.3)	
		Edema	5 (26.3)	
		Confusion	4 (21)	
		Hypokalemia	4 (21)	
		Hyponatremia	4 (21)	
		Drowsiness	2 (10.5)	
		Hypernatremia	1 (5.3)	
		Hyperkalemia	1 (5.3)	
		Nausea	1 (5.3)	
Furosemide – Hydrocortisone (39)	High + High (11)	Fever	7 (63.6)	Serum potassium level and cardiovascular status should be monitored, especially if co-administered. Patients should be advised to inform their physician if they experience potential signs/symptoms of hypokalemia such as constipation, numbness, myalgia, abdominal pain, fatigue, tingling, weakness, irregular heartbeat, muscle cramps, and palpitation.
		Tachycardia	5 (45.4)	
		Constipation	5 (45.4)	
		Confusion	4 (36.4)	
		Irregular heart rate	3 (27.3)	
		Hypokalemia	2 (18.2)	
		Vomiting	1 (9.1)	
	High + Low (11)	Fever	5 (45.4)	
		Confusion	4 (36.4)	
		Constipation	3 (27.3)	
		Hypokalemia	3 (27.3)	
		Tachycardia	2 (18.2)	
		Irregular heart rate	1 (9.1)	
	Low + High (11)	Fever	7 (63.6)	
		Tachycardia	4 (36.4)	
		Constipation	3 (27.3)	
		Hypokalemia	3 (27.3)	
		Irregular heart rate	3 (27.3)	
		Nausea	2 (18.2)	
		Confusion	1 (9.1)	
	Low + Low (6)	Fever	4 (66.7)	
		Tachycardia	4 (66.7)	
		Hypokalemia	3 (50)	
		Irregular heart rate	2 (33.3)	
		Vomiting	1 (16.7)	
Aspirin – Clopidogrel (37)	High + Low (2)	Bradycardia	1 (50)	Monitor patients’ platelets counts and any sign of bleeding. If an adverse effect is noted, the following options may be considered: (a) Decrease the dose of aspirin (b) GIT protection through proton pump inhibitors and patient should be educated about non-prescribed use of analgesics.
		Hypotension	1 (50)	
	Low + Low (35)	Hypotension	14 (40)	
		Tachycardia	9 (26)	
		Increased APTT	9 (26)	
		Drowsiness	7 (20)	
		Increased PT	7 (20)	
		Weakness	6 (17.1)	
		Increased INR	5 (14.3)	
		Decreased platelets	4 (11.4)	
		Palpitations	1 (2.9)	
		Bradycardia	1 (2.9)	
Aspirin – Insulin (33)	Low + High (27)	Tachycardia	11 (41)	Monitoring of patient’s blood glucose and clinical signs of hypoglycemia is suggested. Adjust the dose of insulin if necessary.
		Loss of consciousness	7 (26)	
		Drowsiness	5 (18.5)	
		Pale	3 (11.1)	
		Confusion	2 (7.4)	
		Decreased FBS	2 (7.4)	
		Irritability	1 (3.7)	
		Seizures	1 (3.7)	
		Palpitations	1 (3.7)	
	Low + Low (5)	Pale	1 (20)	
		Tachycardia	1 (20)	
	High + Low (1)	Confusion	1 (100)	
Isoniazid – Rifampin (33)	Low + Low (27)	Fever	22 (81.5)	Monitoring of hepatotoxicity (jaundice, vomiting, fever, anorexia, and LFTs) is advised.
		Anorexia	12 (44.4)	
		Increased ALP	9 (33.3)	
		Vomiting	6 (22.2)	
		Pale	5 (18.5)	
		Anemia	3 (11.1)	
		Weight loss	2 (7.4)	
		Weakness	2 (7.4)	
		Increased ALT	2 (7.4)	
		Increased serum bilirubin	2 (7.4)	
		Epigastric pain	1 (3.7)	
		Hepatic encephalopathy	1 (3.7)	
		Tiredness	1 (3.7)	
	High + High (6)	Fever	6 (100)	
		Increased ALP	3 (50)	
		Anorexia Epigastric pain	2 (33.3)	
			1 (16.7)	
		Weight loss	1 (16.7)	
		Pale	1 (16.7)	
		Increased ALT	1 (16.7)	
		Increased serum bilirubin	1 (16.7)	
Calcium containing products – Ceftriaxone (33)	Low + Low (19)	Fever	11 (57.9)	Ceftriaxone should not be mixed or administered concomitantly with calcium-containing intravenous preparations in the same intravenous administration line. Monitor patient for signs of nephrotoxicity or decreased ceftriaxone effectiveness.
		Increased BUN	10 (52.6)	
		Cough	9 (47.4)	
		Increased serum bilirubin	7 (36.8)	
		Leukocytosis	7 (36.8)	
		Chest pain	2 (10.5)	
	Low + High (7)	Cough	4 (57.1)	
		Fever	3 (42.8)	
		Increased BUN	3 (42.8)	
		Increased serum bilirubin	3 (42.8)	
		Leukocytosis	2 (28.6)	
		Chest pain	1 (14.3)	
	High + Low (5)	Fever	4 (80)	
		Increased BUN	2 (40)	
		Increased serum bilirubin	2 (40)	
		Leukocytosis	2 (40)	
		Chest pain	2 (40)	
		Cough	1 (20)	
		Sepsis	1 (20)	
	High + High (2)	Increased BUN	2 (100)	
		Increased serum bilirubin	2 (100)	
		Leukocytosis	2 (100)	
Pyrazinamide – Rifampin (32)	Low + Low (28)	Fever	23 (82.1)	Monitoring of LFTs during treatment is recommended.
		Anorexia	13 (46.4)	
		Increased ALP	10 (35.7)	
		Vomiting	6 (21.4)	
		Pale	5 (17.8)	
		Anemia Increased serum bilirubin	3 (10.7)	
			2 (7.1)	
		Weakness	2 (7.1)	
		Weight loss	2 (7.1)	
		Increased ALT	1 (3.6)	
		Epigastric pain	1 (3.6)	
		Hepatic encephalopathy	1 (3.6)	
		Tiredness	1 (3.6)	
	High + High (4)	Fever	4 (100)	
		Increased ALP	2 (50)	
		Anorexia	1 (25)	
		Epigastric pain	1 (25)	
		Increased ALT	1 (25)	
		Increased serum bilirubin	1 (25)	
Aspirin – Ramipril (28)	Low + Low (21)	Increased BUN	18 (85.7)	Monitor patients’ blood pressure, hemodynamic parameters, and renal function. Incase of an adverse event, consider the following: (a) replace ACE inhibitors with angiotensin receptor blockers (b) an alternative non-aspirin antiplatelet agent (c) aspirin dosage less than 100 mg per day.
		Increased serum creatinine	12 (57.1)	
		Hypertension	9 (42.8)	
		Tachycardia	8 (38.1)	
		Confused	3 (14.3)	
		Hypokalemia	3 (14.3)	
		Chest pain	2 (9.5)	
		Headache	1 (4.7)	
		Irregular heart rate	1 (4.7)	
	Low + High (7)	Hypertension	5 (71.4)	
		Increased BUN	3 (42.8)	
		Increased serum creatinine	3 (42.8)	
		Tachycardia	2 (28.6)	
		Chest pain	1 (14.3)	
		Hyperkalemia	1 (14.3)	
Albuterol – Furosemide (28)	High + High (9)	Tachycardia	4 (44.4)	Potassium balance and cardiovascular status should be monitored, especially if the beta-2 agonist is administered by nebulizer or systemically. Patients should be advised to inform their physician if they experience potential signs/symptoms of hypokalemia such as constipation, numbness, myalgia, abdominal pain, fatigue, tingling, weakness, irregular heartbeat, muscle cramps, and palpitation.
		Constipation	4 (44.4)	
		Fever	3 (33.3)	
		Hypokalemia	3 (33.3)	
		Confusion	2 (22.2)	
		Vomiting	1 (11.1)	
		Dehydration	1 (11.1)	
	High + Low (17)	Constipation	4 (23.5)	
		Tachycardia	4 (23.5)	
		Hypokalemia	4 (23.5)	
		Confusion	3 (17.6)	
		Vomiting	2 (11.7)	
		Fatigue	2 (11.7)	
		Weakness	2 (11.7)	
		Fever	1 (5.9)	
	Low + High (1)	Constipation	1 (100)	
	Low + Low (1)	Constipation	1 (100)	
Aspirin – Bisoprolol (23)	Low + Low (14)	Hypertension	7 (50)	Patients’ blood pressure and hemodynamic parameters should be monitored.
		Tachycardia	4 (28.5)	
		Chest pain	3 (21.4)	
		Irregular heart rate	2 (14.3)	
		Headache	2 (14.3)	
		Restless	1 (7.1)	
		Drowsiness	1 (7.1)	
	Low + High (7)	Hypertension	6 (85.7)	
		Tachycardia	6 (85.7)	
		Chest pain	3 (42.8)	
		Irregular heart rate	2 (28.6)	
	High + High (1)	Irregular heart rate	1 (100)	
		Headache	1 (100)	
		Hypertension	1 (100)	
		Tachycardia	1 (100)	
	High + Low (1)	–	–	

*ALT* Alanine Aminotransferase, *ACE* Angiotensin Converting Enzyme, *ALP* Alkaline Phosphatase, *APTT* Activated Partial Thromboplastin Time, *BUN* Blood Urea Nitrogen, *FBS* Fasting Blood Sugar, *GIT* Gastrointestinal Tract, *HbA1c* Glycated hemoglobin (A1c), *INR* International Normalized Ratio, *LFTs* Liver Function Tests, *PT* Prothrombin Time, *RBS* Random Blood Sugar

^a^Frequencies were given in round brackets

^b^Adverse outcomes were defined as follows, increased blood urea nitrogen (BUN): BUN ≤20 mg/dL; increased serum creatinine: serum creatinine > 1.06 mg/dL; hypernatremia: serum sodium > 145 mmol/L; hyponatremia: serum sodium < 135 mmol/L; hyperkalemia: serum potassium > 5.5 mmol/L; hypokalemia: serum potassium < 3.5 mmol/L; hyperchloremia: serum chloride > 105 mmol/L; hypertension: systolic blood pressure (BP) > 130 mmHg and/or diastolic BP > 90 mmHg; hypotension: systolic BP < 80 mmHg and/or diastolic BP < 50 mmHg; tachycardia: heart rate > 100 beats/min; bradycardia: heart rate < 70 beats/min; increased activated partial thromboplastin time (APTT): APTT > 35.5 s; increased prothrombin time (PT): PT > 15.5 s; increased international normalized ratio (INR): INR > 1.2; decreased platelets: platelets count < 150,000/μL; hypoglycemia: random blood sugar < 80 mg/dL or fasting blood sugar < 70 mg/dL; increased alkaline phosphatase: > 126 U/L; increased serum bilirubin: > 1 mg/dL; increased alanine aminotransferase: > 59 U/L (male), > 36 U/L (female); leukocytosis: total leukocyte count > 11,000/μL

^c^Percentages were calculated based on dose categories

## Discussion

The issue of drug interactions remains one of the considerable factors among hospitalized patients [7]. This report presents the prevalence, categorization, risk factors, and clinical relevance of pDDIs among hospitalized pneumonia patients. The area remains poorly addressed, locally as well as globally, therefore such studies are needed. The overall prevalence of pDDIs in the current study was higher (73.1%) as compared with that among patients with certain diseases such as HIV (52.2%) [22], liver cirrhosis (21.5%) [23], hypertension (48%) [24], and pediatric patients with respiratory diseases (38.9%) [25]. While, this prevalence of pDDIs is lower in comparison with that among patients with chronic obstructive pulmonary disease (90%) [26] and hemodialysis (89.1%) [27]. Moreover, in our sample, prevalence of major-pDDIs was higher (53.8%) in comparison to that among patients with chronic obstructive pulmonary disease (20% at hospital admission and 24% at hospital discharge) [26], liver cirrhosis (21.4%) [23], hepatitis C (30–44%) [28], and pediatric patients with respiratory diseases (9.5%) [25]. Regardless of variations in the study design, study population, drug utilization/prescribing pattern, consideration of pDDIs types, and drug interaction screening software, our findings indicated higher prevalence of pDDIs. Based on the current findings, patients with pneumonia are at higher risk to DDIs. Therefore, to minimize, prevent, or manage DDIs in hospitals settings following evidence-based strategies have been suggested: use of computerized screening programs for screening medications profiles for pDDIs [29], involving clinical pharmacist for the assessment of pDDIs [11, 13, 30], method for structured evaluation of pDDIs [31], and appraisal of pertinent labs investigations for clinical relevance of interactions [7, 32].

Identifying the type of pDDIs by health care professionals is necessary for the management of adverse events related to pDDIs, reducing/preventing the associated risk, and clinical management of pDDIs. In this study, pDDIs of moderate- and major-severity were frequently identified. Concerning documentation-levels, fair- and good-type were highly prevalent. Similar findings were observed by other studies among hospitalized patients [14, 15, 24]. These findings warrant pneumonia patients to be at risk for the pDDIs associated adverse consequences. Such patients should be monitored for any negative clinical consequences expected due to DDIs.

Polypharmacy is a considerable issue in hospitalized patients with pneumonia [5]. It refers to prescribing more than five drugs at a time [33, 34]. Pneumonia patients receive co-prescription of a number of medicines for the treatment of comorbidities or associated symptoms [5, 6]. A positive relationship of polypharmacy with pDDIs reported by our study is supported by previous published studies [12, 14, 35, 36]. Moreover, in this study separate odds of exposure to major-pDDIs have been calculated. The findings of statistically significant relationship of major-pDDIs with polypharmacy are consistent with findings from previous studies [36, 37]. In addition, we found significant association of major-pDDIs with tuberculosis. The possible reason is prescription of anti-TB drugs among patients with TB and these drugs are responsible for a large number of clinically important DDIs [38]. In this regard, hospitalized patients with pneumonia are at increased risk to pDDIs due to these predictors. Health care professionals should have knowledge regarding all predictors increasing the risk of pDDIs to individualize patients more at risk, optimize medications therapy, and minimize or prevent pDDIs.

PDDIs of any type of severity are not clinically important. So, developing list of clinically important and most frequently observed interactions is of immense need. The list will be used by physicians and pharmacists for the development of therapeutic guidelines and timely/selective identification of pDDIs. A physician’s understanding and knowledge of DDIs can reduce the occurrence of associated adverse events, adjust therapeutic regimen of high-risk patients, provide better quality care, and prevent associated medico-legal concerns. In this study, potential adverse consequences of the most frequent pDDIs were nephrotoxicity, hypokalemia, bleeding, hypoglycemia, hepatotoxicity, reduction in therapeutic effectiveness, hypotension, QT interval prolongation, and hyperkalemia. These findings are somehow consistent with findings of a study on hospitalized patients with liver cirrhosis in which most prevalent potential adverse outcomes due to pDDIs were hyperkalemia, hypoglycemia, renal function deterioration, QT interval prolongation, and bleeding risk [23].

We have related potential adverse effects of DDIs with clinical manifestations of the patients. Such analysis is rarely observed in published literature. Some studies have investigated adverse effects related to interactions but these studies do not specify monitoring parameters and adverse effects for most commonly interacting drug pairs [23, 39]. This is a novel approach that will be helpful for health care professionals to monitor and manage the adverse outcomes related to interactions. This study also considered doses of the administered drugs. Higher doses of the drugs may potentiate the negative consequences of the interactions. As evident from our findings that adverse events were more prevalent among patients prescribed with higher doses as compared to lower doses. Such considerations give more insight in understanding and management of adverse outcomes of interactions. Furthermore, monitoring parameters and management guidelines will support health care professionals regarding proper assessment and management of drug interactions in pneumonia.

### Strengths and limitations

Following are the potential limitations of this study. The pDDIs recorded in this report are mainly associated with the use of medicines for managing several comorbid illnesses or associated signs/symptoms because of hospitalization of pneumonia patients due to these conditions. Therefore, the findings of this study might not be applicable to ambulatory patients because of variable nature/prevalence of disease and drug interactions. Furthermore, we use the term potential DDIs, as, we do not actually observe DDIs. Data are scarce, concerning negative clinical consequences caused by DDIs, but, some retrospective studies are available in the published literature. One study observed increased odds ratios for digoxin toxicity among patients treated with clarithromycin, for hypoglycemia in patients with co-trimoxazole combined with glyburide, and for hyperkalemia among patients who used ACE inhibitors and potassium-sparing diuretics, concurrently [8]. Another study, reported that, there is five times increase risk of sudden death due to cardiac causes, among patients who were treated with erythromycin and CYP3A inhibitors, concurrently [40].

## Conclusions

PDDIs are highly prevalent in patients with pneumonia. Computerized drug interactions screening programs will help in identification, prevention, and minimization of pDDIs in pneumonia patients. Most frequent and clinically important pDDIs need particular attention. Polypharmacy and tuberculosis increase the risk of pDDIs. Identifying patients more at risk to pDDIs and careful monitoring of pertinent signs/symptoms and laboratory investigations are important measures to reduce pDDIs and their related adverse consequences.

## Data Availability

The datasets used and/or analyzed during the current study are available from the corresponding author on reasonable request.

## Abbreviations

ACE: Angiotensin converting enzyme
ADRs: Adverse drug reactions
ALP: Alkaline phosphatase
ALT: Alanine aminotransferase
APTT: Activated partial thromboplastin time
ATD: Alternate day
BD: Twice a day
BP: Blood pressure
BUN: Blood urea nitrogen
CI: Confidence interval
DDIs: Drug-drug interactions
FBS: Fasting blood sugar
GIT: Gastrointestinal tract
HbA1c: Glycated hemoglobin (A1c)
HMC: Hayatabad medical complex
INR: International normalized ratio
IQR: Interquartile range
KTH: Khyber teaching hospital
LFTs: Liver function tests
OD: Once a day
OR: Odds ratios
pDDIs: potential DDIs
PT: Prothrombin time
QID: Four times a day
RBS: Random blood sugar
TDS: Three times a day
